# Supplementary figures and images for: Small molecules restore azole activity against drug-tolerant and drug-resistant Candida isolates
Source: mBio. 2023 Jun 16;14(4):e00479-23. doi: 10.1128/mbio.00479-23 (PMC10470600; doi:10.1128/mbio.00479-23)

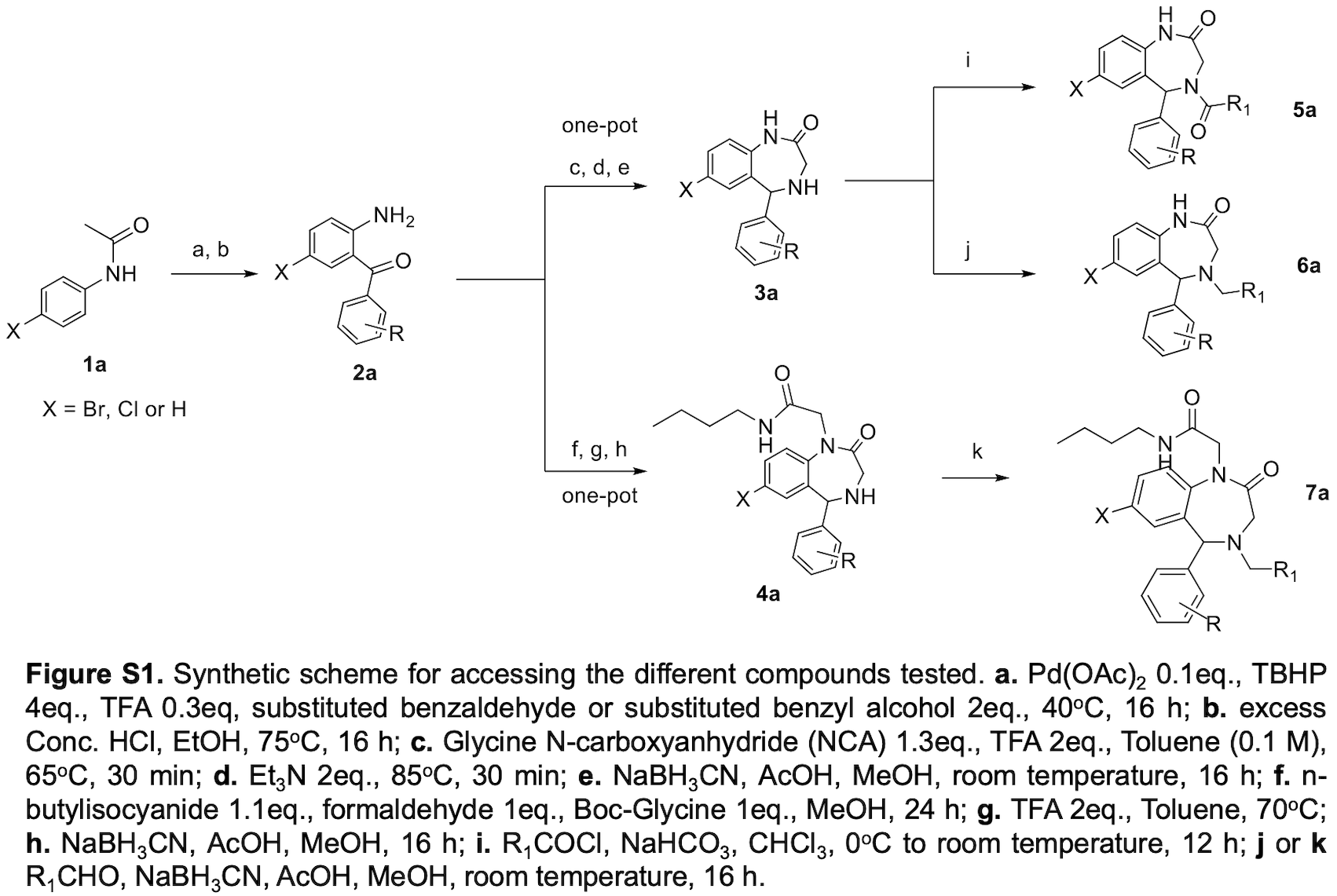

Supplement: Figure S1 — Synthetic scheme for accessing the different compounds tested. [file mbio.00479-23-s0001.tif]

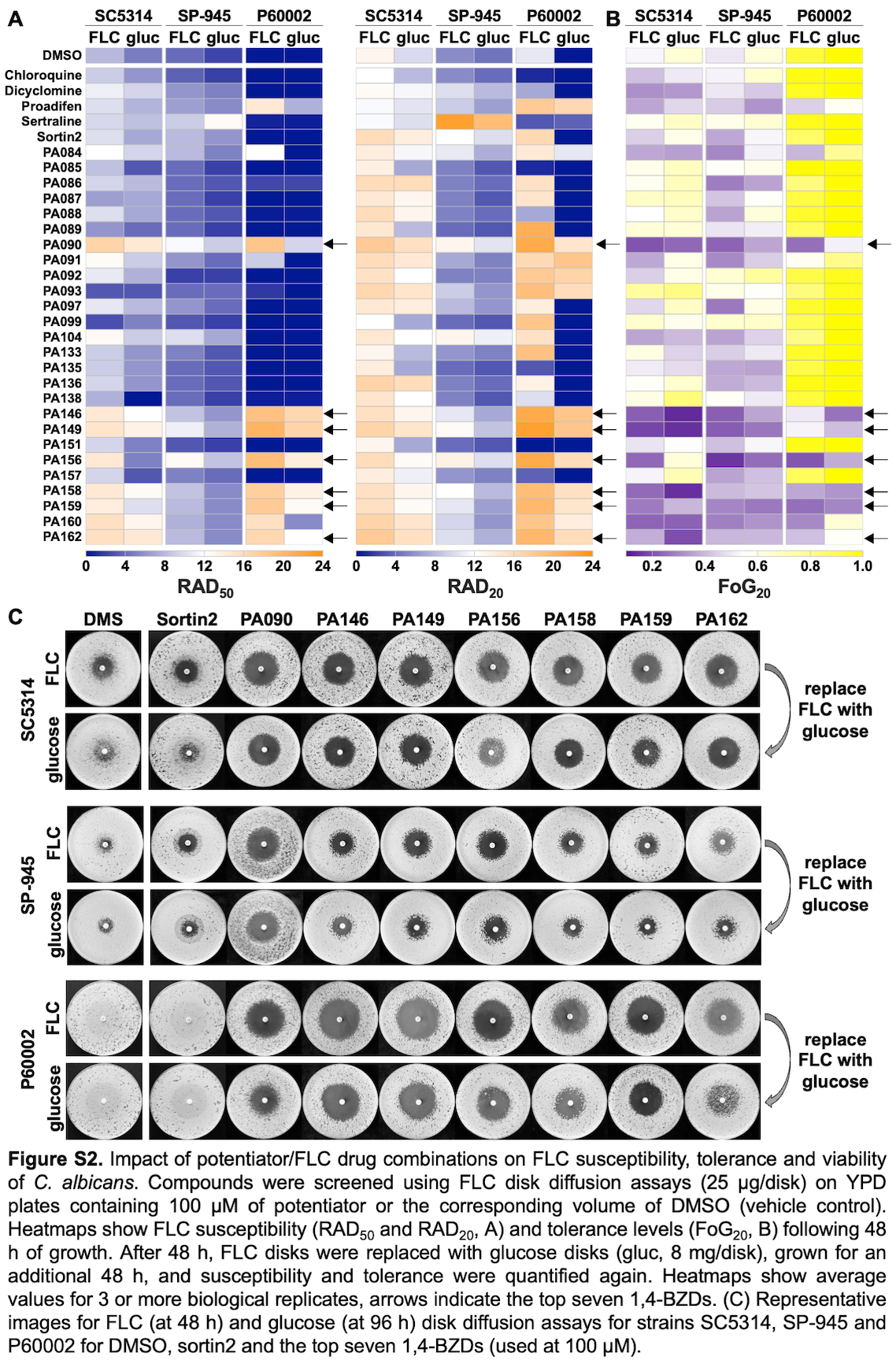

Supplement: Figure S2 — Impact of potentiator/FLC drug combinations on FLC susceptibility, tolerance, and viability of C. albicans. [file mbio.00479-23-s0002.tif]

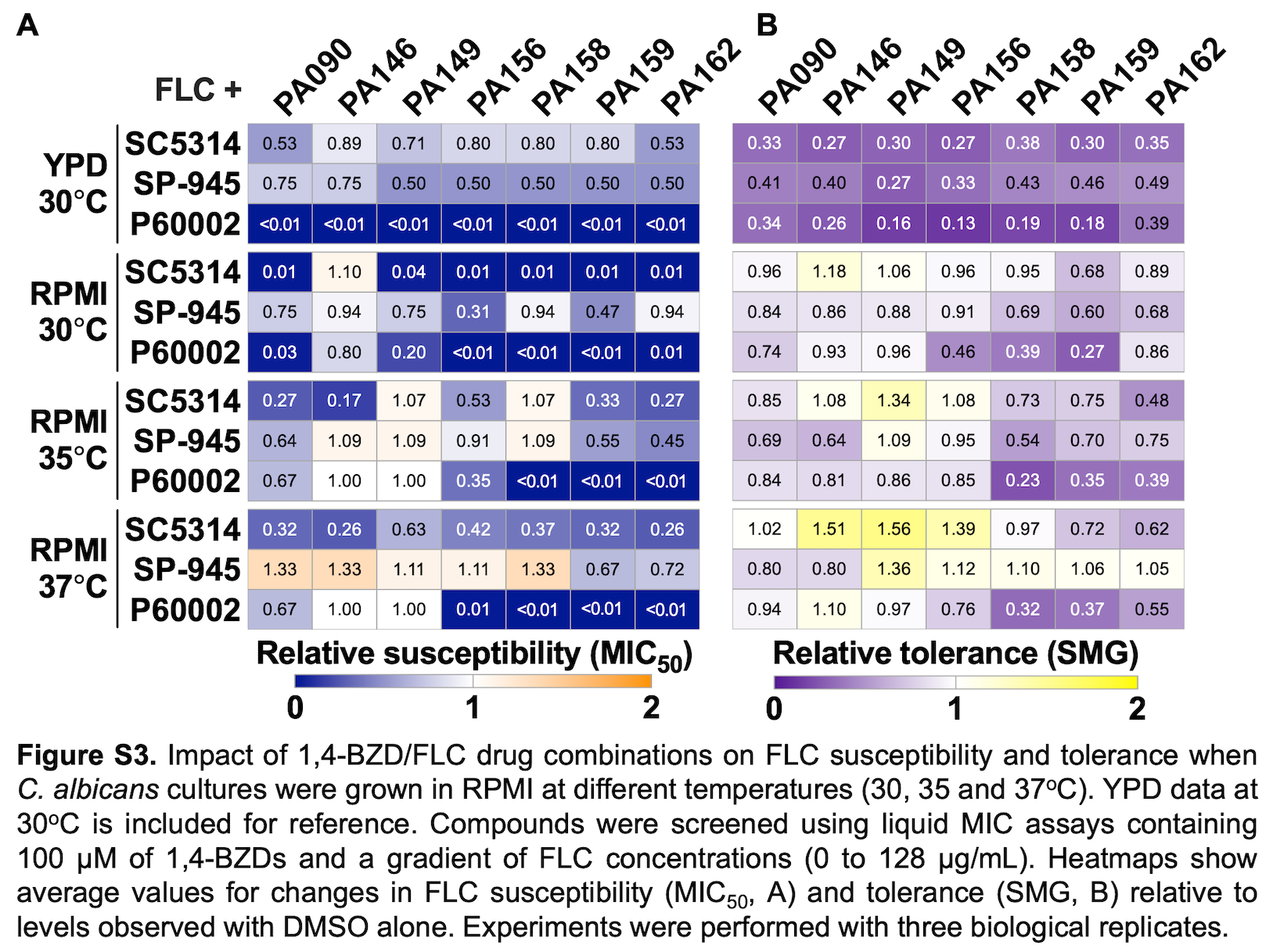

Supplement: Figure S3 — Impact of 1,4-BZD/FLC drug combinations on FLC susceptibility and tolerance when C. albicans cultures were grown in RPMI at different temperatures (30, 35, and 37oC). [file mbio.00479-23-s0003.tif]

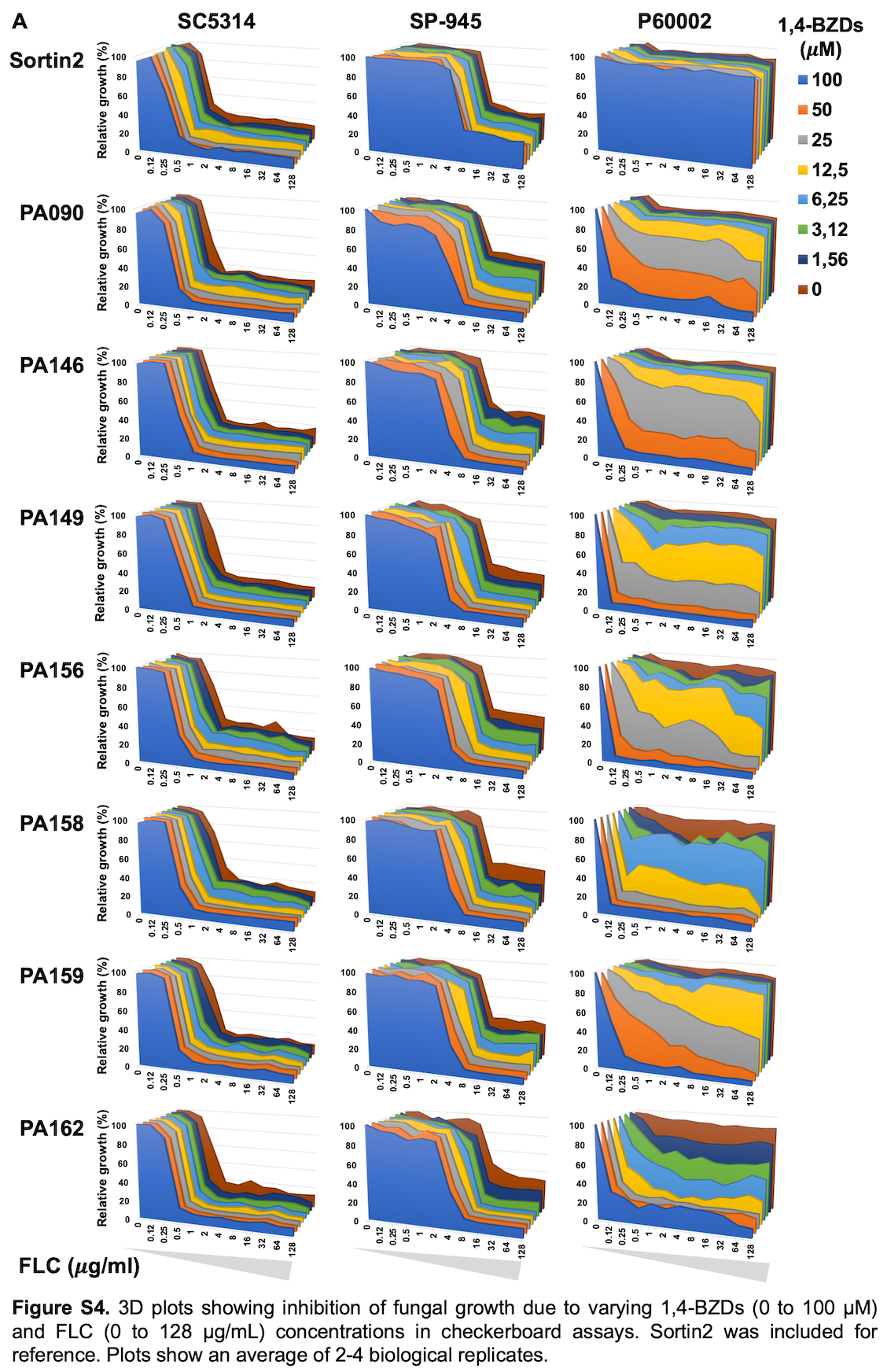

Supplement: Figure S4 — 3D plots showing inhibition of fungal growth due to varying 1,4-BZDs (0 to 100 µM) and FLC (0 to 128 µg/mL) concentrations in checkerboard assays. [file mbio.00479-23-s0004.tif]

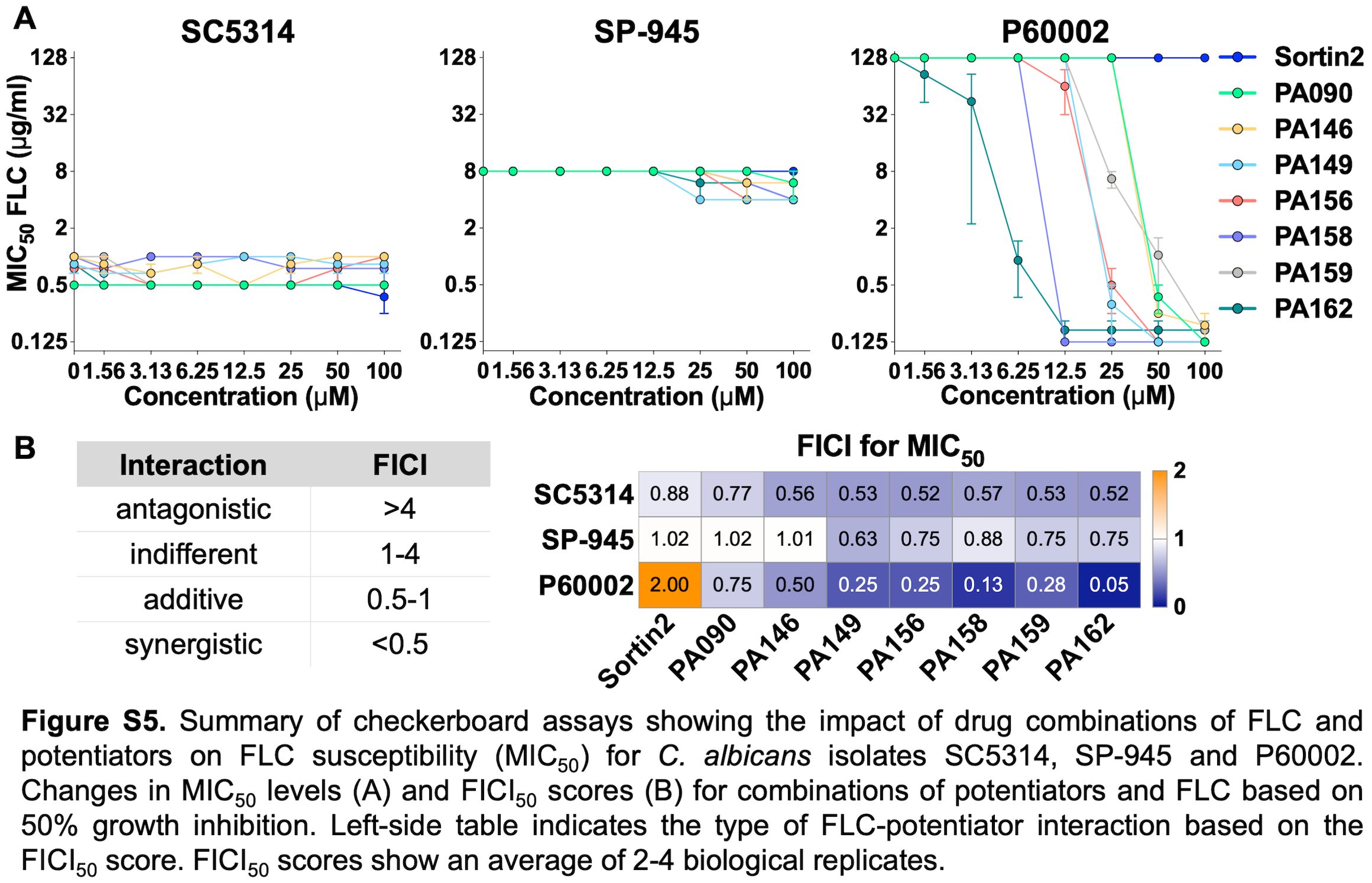

Supplement: Figure S5 — Summary of checkerboard assays showing the impact of drug combinations of FLC and potentiators on FLC susceptibility (MIC50) for C. albicans isolates SC5314, SP-945, and P60002. [file mbio.00479-23-s0005.tiff]

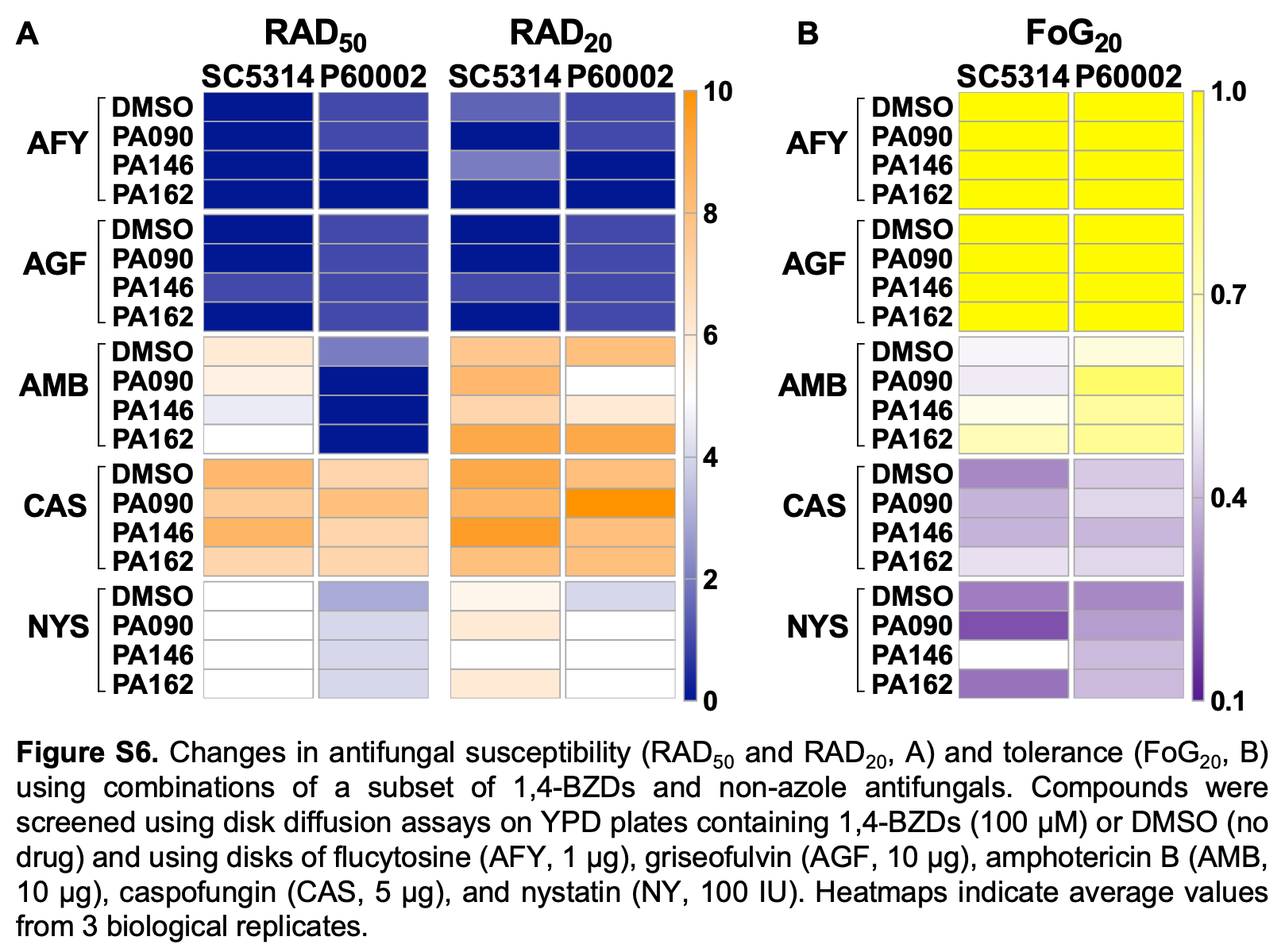

Supplement: Figure S6 — Changes in antifungal susceptibility (RAD50 and RAD20, A) and tolerance (FoG20, B) using combinations of a subset of 1,4-BZDs and non-azole antifungals. [file mbio.00479-23-s0006.tif]

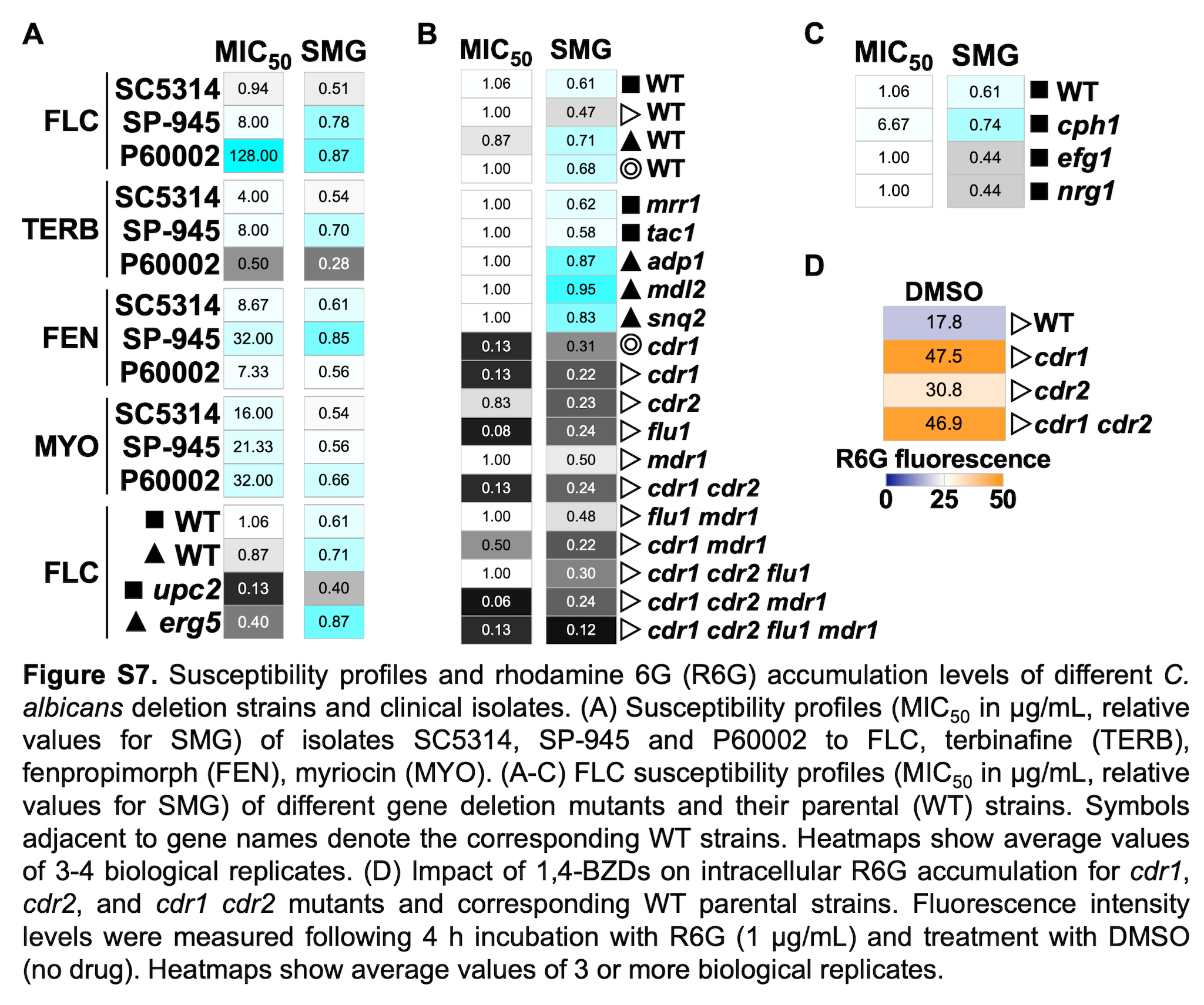

Supplement: Figure S7 — Susceptibility profiles and rhodamine 6G (R6G) accumulation levels of different C. albicans deletion strains and clinical isolates. [file mbio.00479-23-s0007.tiff]

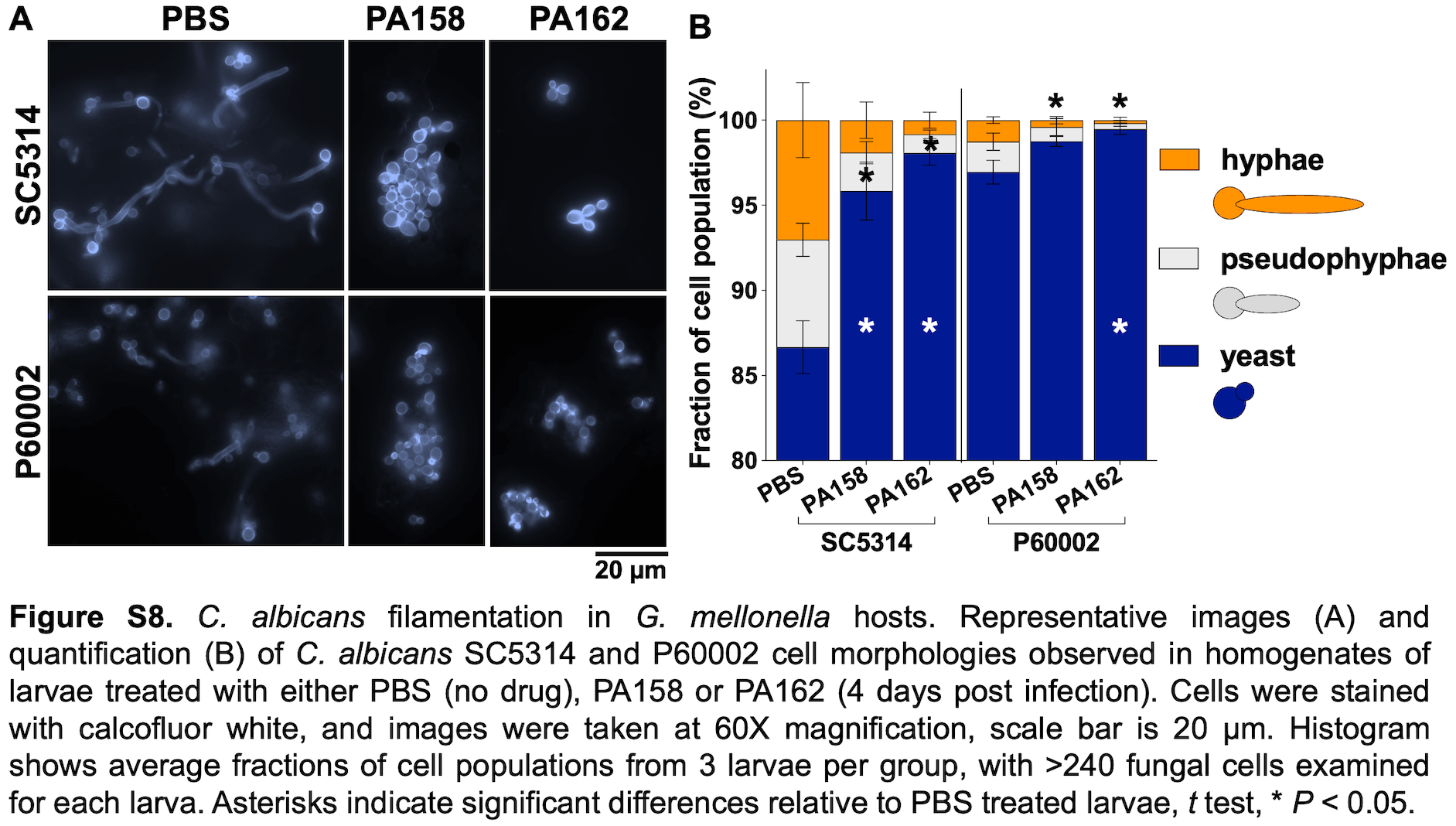

Supplement: Figure S8 — C. albicans filamentation in G. mellonella hosts. [file mbio.00479-23-s0008.tif]
